# Hormesis Responses of Growth and Photosynthetic Characteristics in *Lonicera japonica* Thunb. to Cadmium Stress: Whether Electric Field Can Improve or Not?

**DOI:** 10.3390/plants12040933

**Published:** 2023-02-18

**Authors:** Zhouli Liu, Lei Tian, Mengdi Chen, Luhua Zhang, Qingxuan Lu, Jianbing Wei, Xiangbo Duan

**Affiliations:** 1 Liaoning Key Laboratory of Urban Integrated Pest Management and Ecological Security, College of Life Science and Engineering, Shenyang University, Shenyang 110044, China; 2 Northeast Geological S&T Innovation Center of China Geological Survey, Shenyang 110000, China; 3 Key Laboratory of Mollisols Agroecology, Northeast Institute of Geography and Agroecology, Chinese Academy of Sciences, Changchun 130102, China; 4 Academy of Forest and Grassland Inventory and Planning of National Forestry and Grassland Administration, Beijing 100714, China; 5 State Owned Ying’emen Forest Farm of Qingyuan Manchu Autonomous County, Fushun 113306, China

**Keywords:** hormesis, electric fields, heavy metal, *Lonicera japonica* Thunb., phytoremediation

## Abstract

“Hormesis” is considered a dose–response phenomenon mainly observed at hyperaccumulator plants under heavy metals stress. In this study, the effects of electric fields on hormesis responses in *Lonicera japonica* Thunb. under cadmium (Cd) treatments were investigated by assessing the plant growth and photosynthetic characteristics. Under Cd treatments without electric fields, the parameters of plant growth and photosynthetic characteristics increased significantly when exposed to 5 mg L^−1^ Cd, and decreased slightly when exposed to 25 mg L^−1^ Cd, showing an inverted U-shaped trend, which confirmed that low concentration Cd has a hormesis effect on *L. japonica*. Under electric fields, different voltages significantly promoted the inverted U-shaped trend of the hormesis effect on the plant, especially by 2 V cm^−1^ voltage. Under 2 V cm^−1^ voltage, the dry weight of the root and leaf biomass exposed to 5 mg L^−1^ Cd increased significantly by 38.38% and 42.14%, and the photosynthetic pigment contents and photosynthetic parameters were also increased significantly relative to the control, indicating that a suitable electric field provides better improvements for the hormesis responses of the plant under Cd treatments. The synergistic benefits of the 5 mg L^−1^ Cd and 2 V cm^−1^ electric field in terms of the enhanced hormesis responses of growth and photosynthetic characteristics could contribute to the promoted application of electro-phytotechnology.

## 1. Introduction

With rapid population growth and intensive human activities, large quantities of chemical contaminants, especially heavy metals released into the environment, have recently attracted global attention [1,2]. Heavy metals in soils are mainly derived from metalliferous mining and waste water irrigation, overuse of agricultural fertilizers and pesticides, warfare and military training, and over recent decades, heavy metals have become ubiquitous environmental contaminants all over the world [3,4]. Increasing emissions of heavy metals pose a significant threat to human health, because they may be accumulated in plants, animals or microorganisms and enter into the food chain [5,6,7,8]. Among heavy metals, cadmium (Cd) is one of the most hazardous pollutants and can cause leaf chlorosis, nutritional imbalance, and growth and photosynthesis inhibition [9,10,11,12,13,14,15]. Current methods for remediating Cd-contaminated soils rely primarily on physical and chemical techniques, which have the disadvantages of high operation cost, limited site scope, and complicated operation and may easily bring secondary contaminations or negative environmental effects [16,17]. In contrast, the phytoremediation technology of heavy metal-contaminated soil is widely considered as a promising and sustainable remediation strategy because of its superior characteristics such as being green, low cost and causing less secondary contamination. The key role of phytoremediation technology is hyperaccumulator uptake or the extraction of heavy metals from contaminated soils [18]. It is considered that the concentrations in hyperaccumulators for accumulating heavy metal elements in contaminated soils can reach more than 100 times of those found in non-accumulators [19,20]. Recently, several studies have indicated that electric fields could improve heavy metal accumulation and stimulate seed germination, growth and development of different plants responding to various environmental stresses [21,22,23,24,25]. The combination of hyperaccumulators and electric fields has also been proposed as a new method to promote remediation efficiency [26,27,28,29,30]. However, limited information is available on the effect of electric fields on the growth and photosynthetic characteristics of Cd-hyperaccumulators.

It is known that numerous studies focus on the toxicity of high-dose environmental contaminants [31,32,33,34]; however, some low-dose environmental contaminants may have beneficial effects on organisms [35,36,37]. The beneficial effects of low-dose environmental contaminants is widely recognized in the field of toxicology and medicine, where it is defined as “hormesis”, characterized by a biphasic adaptive response [38,39,40]. It is also found that hormesis can improve the adaptation of plants to some adverse environments, such as the low doses of nitrogen, lanthanum, ozone, ultraviolet radiation and herbicides [41,42,43,44,45,46,47,48]. Some researchers observed that hormesis can protect plants against environmental stress and enhance plant biomass productivity and functional components [49,50,51]. Nevertheless, few studies focus on the relationship of hormesis and hyperaccumulation. Thus, it is very necessary to investigate the hormesis response strategy of hyperaccumulators, especially the electric field-assisted effects on the hormesis responses of growth and photosynthetic characteristics in a hyperaccumulator.

*Lonicera japonica* Thunb. (Japanese honeysuckle) is a popular ornamental plant and has become established in temperate and tropical regions worldwide in the past 150 years [52]. It is commonly cultivated as a highly valued garden plant in urban greening because of its high biomass and easy cultivation, and its deep roots and shoots could reach as long as 150 cm. It also possesses the characteristics of extensive competitive ability, wide geographic distribution, and strong resistance to environmental stresses such as bacterial, viral and oxidative interference [53]. Our previous studies showed that *L. japonica* has a strong tolerance and good accumulation capability for Cd in plant tissues (the stem and shoot Cd accumulated concentrations in *L. japonica* can reach 344.49 ± 0.71 and 286.12 ± 9.38 μg g^−1^ DW, respectively), and it is recognized as a new woody ornamental Cd-hyperaccumulator [9,54,55]. Moreover, we also found that the growth, photosynthetic pigment and relative water contents in *L. japonica* were stimulated by hormesis under low concentrations of Cd stress [9,54,55,56,57]. In the present study, we selected *L. japonica* as a model plant to investigate the effect of different electric fields on the hormesis responses of the growth, photosynthetic pigment composition and photosynthesis of the plant. The specific objectives are to explore whether an electric field can improve the hormesis responses of the plant under different concentrations of Cd stress. It will contribute to a better understanding of the hormesis response strategy of hyperaccumulators and promote the application of electro-phytotechnology.

## 2. Materials and Methods

### 2.1. Plant Materials and Experimental Treatments

The experiment was carried out in a greenhouse of Shenyang Agricultural University (41°44′ N and 123°27′ E, 44.7 m a.s.l.). Seedlings of *L. japonica* were collected from a non-contaminated experimental field and cultivated in sterilized sand by culture medium. The culture medium was Hoagland solution containing the following composition (mmol L^−1^): Ca(NO_3_)_2_·4H_2_O 5.00, MgSO_4_·7H_2_O 2.00, KNO_3_ 5.00, KH_2_PO_4_ 1.00, H_3_BO_3_ 0.05, ZnSO_4_·7H_2_O 0.80 × 10^−3^, MnCl_2_·4H_2_O 9.00 × 10^−3^, CuSO_4_·5H_2_O 0.30 × 10^−3^, (NH_4_)_6_Mo_7_O_24_·4H_2_O 0.02 × 10^−3^, Fe-EDTA 0.10 [54,58]. The pH was measured by a pH meter and the pH value was 5.8 ± 0.1.

After the plants were cultivated for 8 weeks, CdCl_2_·2.5H_2_O (Kermel Chemical Reagent Co., Ltd., Tianjin, China, >99%) was added into the culture medium and the Cd treatments were 0, 5 and 25 mg L^−1^. Subsequently, an electric field (EF), which contains a pair of graphite electrodes (10.0 cm in length) associated with a DC power supply (220 V, 50 Hz), was daily 6 h and referred to 0, 1, 2 and 3 V cm^−1^ according to Liu et al. (2022) [59]. The EF-Cd treatments are shown in Table 1. The experiment consisted of three independent replicates. After one week, the plants were harvested for analysis.

### 2.2. Measurements of Plant Biomass and Cd Content

The harvested plants were washed with tap water, and the roots of the plants were immersed in 20 mM Na_2_-EDTA for 15 min and then washed with deionized water to remove Cd adhering to the root surface [9]. The plants were separated into leaves and roots. These plant tissues were dried at 105 °C for 20 min, then at 70 °C until the weight was constant. Subsequently, the dry weight (g) of the root and leaf biomass was obtained.

Dried plant materials were ground to fine powder by a grinder. The powders were digested with a concentrated acid mixture of HNO_3/_HClO_4_ (3:1, *v*/*v*). The plant Cd concentrations in *L. japonica* were determined with a flame atomic absorption spectrophotometer (Perkin-Elmer, Waltham, MA, USA) after microwave digestion.

### 2.3. Deteremination of Photosynthetic Pigment Contents

The photosynthetic pigments were measured by the uniform and similar leaf samples. The leaf samples (0.2 g) were cut into small pieces, and then soaked in 25 mL 95% (*v*/*v*) ethanol at 4 °C in darkness until the tissues became white. The extracting solution absorbance at 649, 665 and 470 nm was measured. The contents of chlorophyll a (Chla), chlorophyll b (Chlb), total chlorophyll (Total Chl) and carotenoid (Car) were calculated by a modified method according to Lichtenthaler and Wellburn (1983) and Lichtenthaler (1987) [60,61].

### 2.4. Assays of Photosynthetic Parameters

The photosynthetic parameters were determined by a portable photosynthesis system (LI-6400, Li-Cor Inc. Lincoln, NE, USA) under different treatments. The photosynthetic parameters consisted of net photosynthetic rate (Pn), stomatal conductance (Gs), transpiration rate (Tr) and intercellular CO_2_ concentration (Ci). During different treatments, the parameters inside the leaf chamber (light level, CO_2_ concentration and leaf temperature) were maintained constant at 1000 μmol m^−2^ s^−1^ PPFD, 25 ± 0.3 °C and 380 ± 5 μmol CO_2_ mol^−1^. The upper second fully expanded leaves were used for the determination according to the method of Pandey et al. (2003) [62].

### 2.5. Statistical Analyses

All measurements in the study were replicated three times. The data analyses were performed as the means ± SD. The statistical analysis of variance was carried out with SPSS 22.0. The significant difference was presented at the *p* < 0.05 level. The least significant difference (LSD) test was used to determine the multiple comparison between treatments.

## 3. Results and Discussion

### 3.1. Effects of Different EF-Cd Treatments on Plant Cd Concentrations

**Figure 1:**
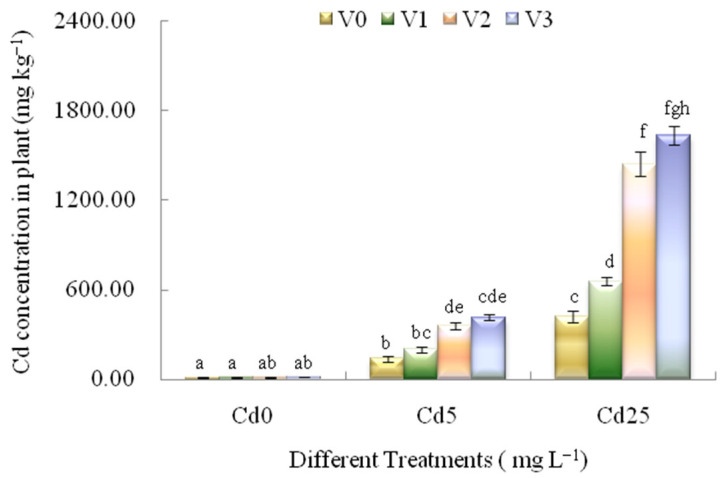
Effects of different EF-Cd treatments on plant Cd concentrations in *L. japonica*. V0, V1, V2 and V3 showed 0, 1, 2 and 3 V cm^−1^ electric fields. Cd0, Cd5, and Cd25 showed 0, 5 and 25 mg L^−1^ Cd treatments. Different letters indicate significant differences at the *p* < 0.05 level. Values represent mean ± SD.

The Cd accumulations in *L. japonica* under different treatments were shown in Figure 1. With the increase of Cd concentrations in the medium without an electric field (T_1_–T_3_, Table 1), the plant Cd concentrations in *L. japonica* had an increased trend, which ranged from 11.11, 137.43 to 419.05 mg kg^−1^. Under the T_4_–T_6_ treatments (V1-Cd0, V1-Cd5 and V1-Cd25), a slight increase of plant Cd concentrations in *L. japonica* under different concentrations by Cd stress was observed by 1 V cm^−1^ voltages, which ranged from 12.31, 198.92 to 654.65 mg kg^−1^. Under the T_7_–T_9_ treatments (V2-Cd0, V2-Cd5 and V2-Cd25), the plant Cd concentrations in *L. japonica* under different concentrations by Cd stress had a significant increased trend compared with the T_1_–T_3_ treatments (under Cd treatments without electric field), which ranged from 13.23, 358.30 to 1440.00 mg kg^−1^. Under the T_10_–T_13_ treatments (V3-Cd0, V3-Cd5 and V3-Cd25), the plant Cd concentrations in *L. japonica* under different concentrations by Cd stress had a more significant increase compared with the T_1_–T_3_ treatments (under Cd treatments without electric field), which ranged from 13.99, 414.58 to 1630.84 mg kg^−1^. It was demonstrated that the concentrations of several heavy metals (Cd, Cu, Zn and Pb) in plants were promoted because of the application of electric fields [27,63]. In the present study, the electric fields significantly enhanced the plant Cd concentrations in *L. japonica* exposed to different concentrations Cd compared with T_1_–T_3_ treatments (under Cd treatments without electric fields). The significant increase of plant Cd concentrations under the electric field were observed when the plants were exposed to different concentrations of Cd, especially exposed to high concentrations (25 mg L^−1^) Cd. Under different concentrations of Cd treatments, the plant Cd concentrations in *L. japonica* were increased significantly by 2 V cm^−1^ voltage and 3 V cm^−1^ voltage, which reached 1440.00 (T_9_, V2-Cd25) and 1630.84 mg kg^−1^ (T_12_, V3-Cd25), which were 3.44 and 3.89 times of the T_3_ treatment (V0-Cd25), respectively. The positive effect of the electric field may be correlated with the variety of metal ions polarity and cell membrane properties in plants [64,65]. The similar results have been reported by Klink et al. (2019) and Yuan et al. (2021), which mainly resulted from the electric field-induced increase of the membrane polarization rate, cell metabolism and activated ion channels [25,66].

### 3.2. Effect of Different EF-Cd Treatments on Plant Growth

**Figure 2:**
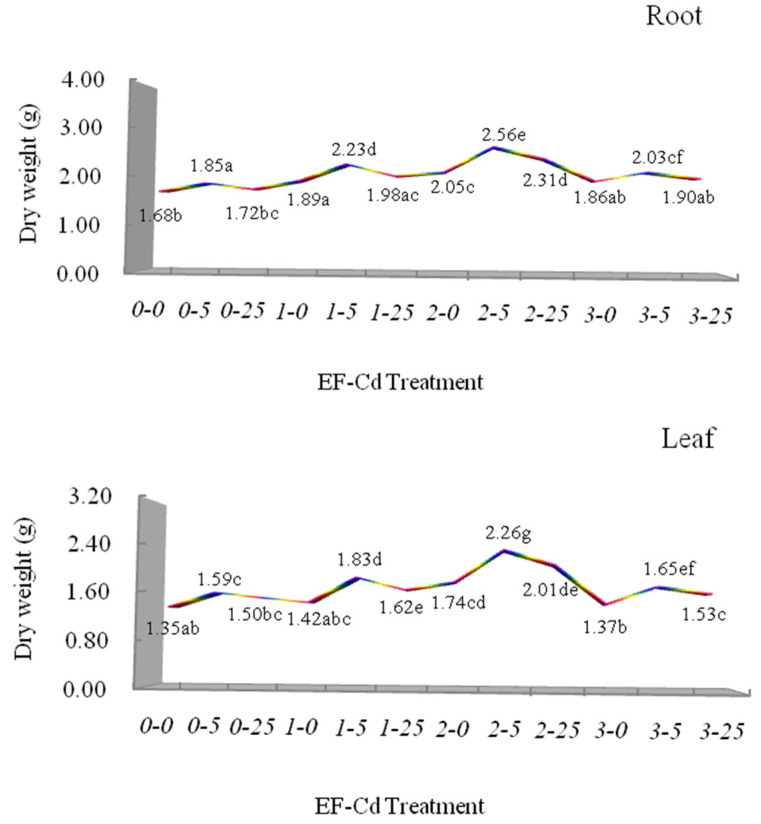
Effects of different EF-Cd treatments on dry weight of root and leaf biomass in *L. japonica*. Different colors showed the different responses in *L*. *japonica* under EF-Cd treatments. Different letters indicate significant differences at the *p* < 0.05 level. Values represent mean ± SD.

It is well known that the biomass of plants is an important highly sensitive indicator responding to heavy metal or other abiotic stresses [32,33]. The growth responses in the form of dry weight of the root and leaf biomass in *L. japonica* under different treatments is displayed in Figure 2. Under the T_1_–T_3_ treatments (under Cd treatments without electric fields), the dry weight of the root biomass exposed to 5 mg L^−1^ Cd increased significantly by 10.12% relative to the T_1_ treatment (V0-Cd0), and had a slight decrease when exposed to 25 mg L^−1^ Cd, indicating an inverted U-shaped curve, which confirmed that low concentration Cd has the hormesis effect on the root growth of *L. japonica*. The results correspond to our previous studies, which showed that the growth characteristics, photosynthetic pigments contents, relative water contents and other physiological parameters of Cd treatments all significantly indicated an inverted U-shaped dose–response curve, confirming that the hormesis effect of low concentration Cd occurred in *L. japonica* [9,54,55,56,57]. Under the T_4_–T_12_ treatments (under electric field), the dry weight of root biomass had an increased trend compared with the T_1_–T_3_ treatments (under Cd treatments without electric fields), which showed that different voltages significantly promoted the inverted U-shaped trend of dry weight of root biomass in *L. japonica*, especially by 1 V cm^−1^ voltage and 2 V cm^−1^ voltage. Under 1 V cm^−1^ voltage and 2 V cm^−1^ voltage, the dry weight of root biomass exposed to 5 mg L^−1^ Cd increased significantly by 20.54% and 38.38% relative to the T_2_ treatment (V0-Cd5), which investigated that the medium voltage (2 V cm^−1^) has more improvements to the hormesis effect of low concentration Cd on the plant growth of *L. japonica*. He et al. (2017) have also reported that the dry weight of root biomass in maize under a drought environment was enhanced by a pulsed electric field, which could be derived from the improvement of the respiration metabolism and substance transformation through the pulsed electric field [67]. Under different voltages, the dry weight of root biomass exposed to 25 mg L^−1^ Cd increased by 15.12%, 34.30% and 10.47% relative to the T_3_ treatment (V0-Cd25), which confirmed our previous study, indicating the electric field-enhanced tolerance of *L. japonica* responded to high concentrations of Cd.

In contrast, under the T_1_–T_3_ treatments (under Cd treatments without electric fields), the dry weight of leaf biomass exposed to 5 mg L^−1^ Cd increased significantly by 17.78% relative to the T_1_ treatment (V0-Cd0), and decreased slightly when exposed to 25 mg L^−1^ Cd, showing a similar inverted U-shaped trend of the hormesis effect with the dry weight of root biomass. Wiewiórka (2013) observed that a high-intensity electric field had limited impacts on the growth of tomatoes in a hydroponic culture [64]. However, in the present study, under the T_4_–T_12_ treatments (under electric field), the dry weight of leaf biomass had an increased trend compared with the T_1_–T_3_ treatments (under Cd treatments without electric field), which indicated that different voltages significantly promoted the inverted U-shaped trend of the dry weight of leaf biomass in *L. japonica*, especially by 1 V cm^−1^ voltage and 2 V cm^−1^ voltage. Under 1 V cm^−1^ voltage and 2 V cm^−1^ voltage, the dry weight of leaf biomass exposed to 5 mg L^−1^ Cd increased significantly by 15.09% and 42.14% relative to the T_2_ treatment (V0-Cd5). Compared with the results of the dry weight of root biomass above, it indicated that the medium voltage (2 V cm^−1^) more significantly enhanced the hormesis effect of low concentration Cd on the leaf biomass than the root biomass in *L. japonica,* which could be the reason that plant organs in *L. japonica* have different sensitivity and tolerance mechanisms when responding to environmental stress. In summary, a medium strength electric field (2 V cm^−1^) could improve the hormesis responses of plant growth in *L. japonica* under different treatments. This is in accordance with those earlier studies that reported that the electric field stimulated the plant growth and productivity though regulating the different levels of plant growth hormones [22,64,66].

### 3.3. Effect of Different EF-Cd Treatments on Photosynthetic Pigment Composition

**Figure 3:**
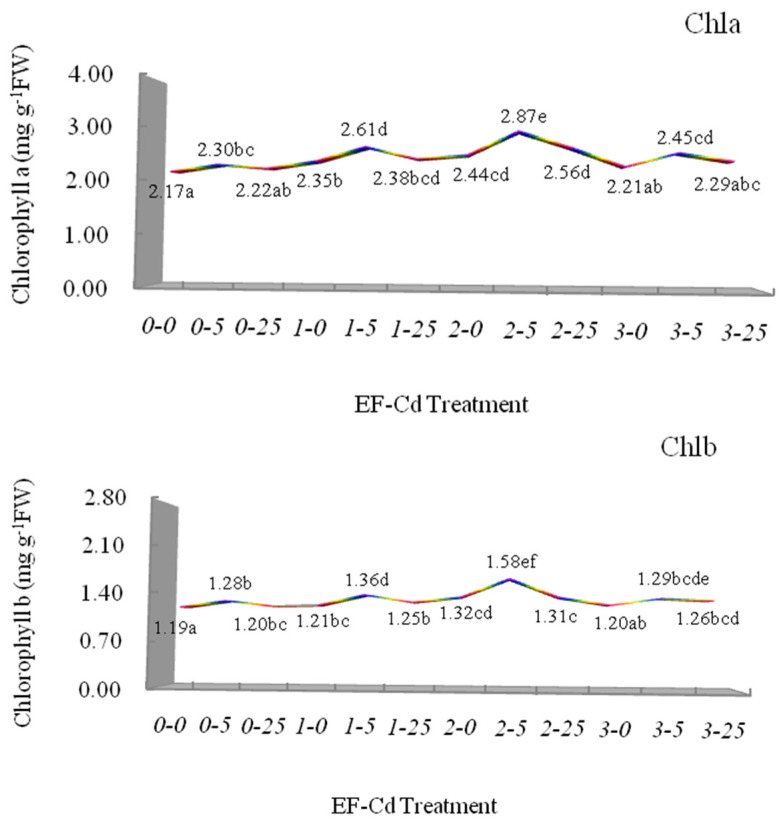

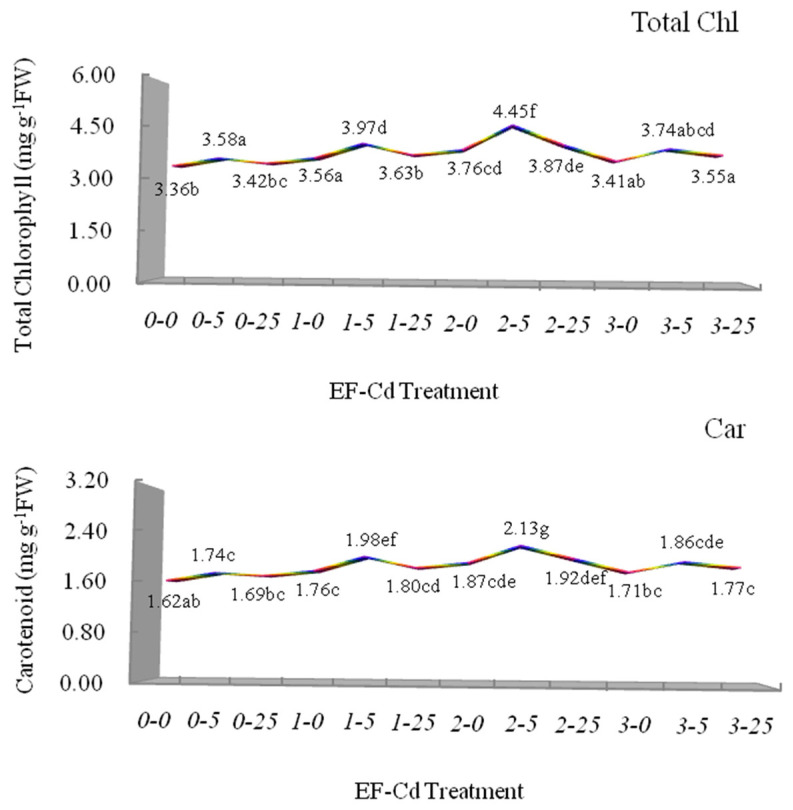
Effects of different EF-Cd treatments on the contents of Chlorophyll a (Chla), Chlorophyll b (Chlb), Total Chlorophyll (Chl) and Carotenoid (Car) in leaves of *L*. *japonica*. Different colors showed the different responses in *L*. *japonica* under EF-Cd treatments. Different letters indicate significant differences at the *p* < 0.05 level. Values represent mean ± SD.

The measured results of photosynthetic pigment composition including chlorophyll a (Chla), chlorophyll b (Chlb), total chlorophyll (Total Chl) and carotenoid (Car) in leaves of *L*. *japonica* are presented in Figure 3. Under the T_1_–T_3_ treatments (under Cd treatments without electric fields), the contents of Chla, Chlb, Total Chl and Car exposed to 5 mg L^−1^ Cd increased significantly by 5.99%, 7.56%, 6.55% and 7.41% relative to T_1_ treatment (V0-Cd0), and had a decrease exposed to 25 mg L^−1^ Cd, which showed an inverted U-shaped curve, indicating low concentration Cd has the hormesis effect on the photosynthetic pigment composition of *L. japonica*. The results confirmed that low concentration Cd could have a stimulatory effect on plant growth, the reasons of which may be the promoted dry matter accumulation and seedling biomass through the increased photosynthetic pigment contents [68,69,70]. When *L. japonica* was exposed to 25 mg L^−1^ Cd without electric fields (T_1_–T_3_ treatments), the contents of Chla, Chlb, Total Chl and Car in the plant showed the decreased trend, which could have resulted from the substitution of chlorophyll Mg^2+^ in photosynthetic pigment composition by Cd^2+^ [71]. With the increase of Cd concentrations in the medium, more chlorophyll Mg^2+^ in the photosynthetic pigment composition are replaced spontaneously by Cd^2+^ and cause the degradation of photosynthetic pigments and even the inhibition of photosynthesis. Under the T_4_–T_12_ treatments (under electric fields), the contents of Chla, Chlb, Total Chl and Car had an increased trend compared with the T_1_–T_3_ treatments (under Cd treatments without electric fields), which showed the electric field could improve the Cd-induced degradation of photosynthetic pigments and stimulate the protective mechanism in *L. japonica*. It was observed that the different voltages significantly promoted the inverted U-shaped trend of the contents of Chla, Chlb, Total Chl and Cars, especially by 1 V cm^−1^ voltage and 2 V cm^−1^ voltage. Moreover, different photosynthetic pigments have different sensibilities to environmental stress [72]. Under 1 V cm^−1^ voltage, the contents of Chla, Chlb, Total Chl and Cars exposed to 5 mg L^−1^ Cd increased significantly by 13.48%, 6.25%, 10.89% and 13.79% relative to the T_2_ treatment (V0-Cd5); by comparison, under 2 V cm^−1^ voltage, the contents of Chla, Chlb, Total Chl and Cars exposed to 5 mg L^−1^ Cd increased significantly by 24.78%, 23.44%, 24.30% and 22.41% relative to the T_2_ treatment (V0-Cd5), which indicated that a medium voltage (2 V cm^−1^) better promotes the hormesis effect of low concentration Cd on the photosynthetic pigment composition of *L. japonica*. The phenomenon is in agreement with the hormesis responses of plant growth in *L. japonica* under different treatments, which mainly result from electric field-induced uptake increase of Fe, Mg or other trace elements [32,73]. When the increased voltage reached 3 V cm^−1^ under the electric field, the contents of Chla, Chlb, Total Chl and Cars exposed to 5 mg L^−1^ Cd had no significant increases relative to the T_2_ treatment (V0-Cd5), the contents of which were 2.45 mg g ^−1^FW, 1.29 mg g ^−1^FW, 3.74 mg g ^−1^FW and 1.86 mg g ^−1^FW, respectively. The results indicated that a suitable electric field could have better improvement for the hormesis responses of photosynthetic pigment composition in *L. japonica* to Cd stress.

### 3.4. Effect of Different EF-Cd Treatments on Photosynthetic Parameters

Photosynthesis, as the basis of all plant growth and crop yield, is undoubtedly the most important biological process and is very susceptible to environments contaminated by Cd [74]. The photosynthesis responses in terms of the net photosynthetic rate (Pn), stomatal conductance (Gs), transpiration rate (Tr) and intercellular CO_2_ concentration (Ci) in *L. japonica* under different treatments are evaluated in Table 2. Under the T_1_–T_3_ treatments (under Cd treatments without electric fields), when the plants were exposed to low concentration (5 mg L^−1^) Cd, the contents of Pn, Gs, Tr and Ci in *L. japonica* had a significant increase compared with the T_1_ treatment (V0-Cd0), which were 15.85 ± 0.91 μmol m^−2^ s^−1^, 0.38 ± 0.01 mol m^−2^ s^−1^, 2.52 ± 0.05 mmol m^−2^ s^−1^ and 383.25 ± 10.78 μL L^−1^, respectively. It is shown that the significant hormesis effect on Pn promoted the gas exchange and transpiration in *L. japonica* in the form of the increased Gs, Tr and Ci, the reasons of which may result from the stimulating impact of low concentration Cd on the Rubisco contents [74]. Under the T_4_–T_12_ treatments (under electric fields), when the plants were exposed to low concentration (5 mg L^−1^) Cd, the contents of Pn, Gs, Tr and Ci in *L. japonica* were all increased significantly by 1 V cm^−1^ voltage (T_5_, V1-Cd5), 2 V cm^−1^ voltage (T_8_, V2-Cd5) and 3 V cm^−1^ voltage (T_11_, V3-Cd5), respectively. Under the T_4_–T_12_ treatments (under electric fields), different voltages significantly promoted the inverted U-shaped trend of the contents of Pn, Gs, Tr and Ci, especially by 2 V cm^−1^ voltage. Under different EF-Cd treatments, the maximum value of Pn, Gs and Tr reached 22.95 ± 0.98 μmol m^−2^ s^−1^, 1.19 ± 0.05 mol m^−2^ s^−1^ and 3.33 ± 0.08 mmol m^−2^ s^−1^, and under low concentration (5 mg L^−1^) Cd treatment, the contents of Pn, Gs, Tr and Ci were all increased significantly by 2 V cm^−1^ voltage (T_8_, V2-Cd5). This is in agreement with the dry weight of root and leaf biomass, which showed that the combination of low concentration (5 mg L^−1^) Cd and medium voltage (2 V cm^−1^) was useful to improve the photosynthesis capacity and plant growth. The photosynthesis responses, in terms of Pn, Gs, Tr and Ci in *L. japonica* under different treatments, also have a good correlation with the change trend of the photosynthetic pigment composition. Several researchers observed that Cd stress had a negative impact on plant photosynthesis, which is probably traceable in the decreased chlorophyll biosynthesis and thylakoids or the inhibited plant growth [68,75,76,77]. However, in the present study, under high concentration (25 mg L^−1^) Cd treatment, the contents of Pn, Gs, Tr and Ci in *L. japonica* were promoted significantly by electric fields relative to the T_3_ treatment (V0-Cd25), which is probably associated with the adaptive mechanisms of hyperaccumulators responding to external stress [78,79,80].

## 4. Conclusions

Based on the previous study, it is shown that *L. japonica* is a good model plant to investigate the effect of different electric fields on the hormesis responses of the growth, photosynthetic pigment composition and photosynthesis of plants. In the study, under the T_1_–T_3_ treatments (under Cd treatments without electric fields), the parameters of plant growth (dry weight of root and leaf biomass), photosynthetic pigment composition (Chla, Chlb, Total Chl and Cars) and photosynthesis (Pn, Gs, Tr and Ci) increased significantly when exposed to 5 mg L^−1^ Cd (*p* < 0.05), and had a slight decrease when exposed to 25 mg L^−1^ Cd, showing an inverted U-shaped trend, which confirmed that low concentration Cd has a hormesis effect on *L. japonica*. Under the T_4_–T_12_ treatments (under electric field), different voltages significantly promoted the inverted U-shaped trend of the hormesis effect, especially by 2 V cm^−1^ voltage, which indicated that a suitable electric field better improves the hormesis responses of growth photosynthetic pigment composition and photosynthesis in *L. japonica* to Cd stress. The present results will be useful to explore the underlying mechanisms of the hormesis effect of Cd stress on hyperaccumulators for electric field-assisted phytoremediation.

## Figures and Tables

**Table 1:** The treatments in the study.

Different Treatments	EF-Cd Treatment	Electric Field (V cm^−1^)	Cd Treatment (mg L^−1^)
T_1_	0-0	0 (V0)	0 (Cd0)
T_2_	0-5	0(V0)	5 (Cd5)
T_3_	0-25	0 (V0)	25 (Cd25)
T_4_	1-0	1 (V1)	0 (Cd0)
T_5_	1-5	1 (V1)	5 (Cd5)
T_6_	1-25	1 (V1)	25 (Cd25)
T_7_	2-0	2 (V2)	0 (Cd0)
T_8_	2-5	2 (V2)	5 (Cd5)
T_9_	2-25	2 (V2)	25 (Cd25)
T_10_	3-0	3 (V3)	0 (Cd0)
T_11_	3-5	3 (V3)	5 (Cd5)
T_12_	3-25	3 (V3)	25 (Cd25)

**Table 2:** Effect of different EF-Cd treatments on photosynthetic parameters in *L*. *japonica*.

Different Treatments	Pn (μmol m^−2^ s^−1^)	Gs (mol m^−2^ s^−1^)	Tr (mmol m^−2^ s^−1^)	Ci (μL L^−1^)
T_1_	13.61 ± 0.45 a	0.16 ± 0.02 ab	1.77 ± 0.10 a	357.23 ± 8.15 a
T_2_	15.85 ± 0.91 b	0.38 ± 0.01 d	2.52 ± 0.05 bc	383.25 ± 10.78 b
T_3_	14.26 ± 0.36 c	0.22 ± 0.02 bc	2.38 ± 0.03 d	320.92 ± 16.51 cd
T_4_	15.65 ± 0.83 ab	0.36 ± 0.02 d	1.97 ± 0.07 ab	337.51 ± 11.02 c
T_5_	19.72 ± 0.42 d	0.77 ± 0.04 e	2.91 ± 0.04 e	342.97 ± 18.65 abc
T_6_	16.33 ± 0.85 abc	0.43 ± 0.02 bcd	2.59 ± 0.12 cd	341.86 ± 8.97 ab
T_7_	17.62 ± 0.51 bc	0.56 ± 0.03 cde	2.17 ± 0.06 d	316.45 ± 13.52 cd
T_8_	22.95 ± 0.98 ef	1.19 ± 0.05 fg	3.33 ± 0.08 gh	375.08 ± 19.33 b
T_9_	18.74 ± 0.72 cd	0.67 ± 0.03 ef	2.83 ± 0.11 def	365.63 ± 9.82 bc
T_10_	14.13 ± 0.69 a	0.21 ± 0.02 b	1.82 ± 0.09 ab	281.36 ± 17.20 ef
T_11_	17.45 ± 0.57 cde	0.54 ± 0.01 de	2.68 ± 0.06 def	320.27 ± 11.26 cd
T_12_	15.56 ± 0.62 b	0.35 ± 0.03 abc	2.51 ± 0.05 c	333.29 ± 14.91 bcd

Data are means ± SD. Different letters indicate significant differences at the *p* < 0.05 level. Pn: net photosynthetic rate; Gs: stomatal conductance; Tr: transpiration rate; Ci: intercellular CO_2_ concentration.

## Data Availability

The data presented in the study are available on request from the corresponding author. The data are not publicly available due to the restriction policy of the coauthors’ affiliations.
